# Comparison of Clinical Characteristics and Biomarkers in Culture-Positive and Culture-Negative Sepsis Patients

**DOI:** 10.7759/cureus.58682

**Published:** 2024-04-21

**Authors:** Pawan K Goyal, Shruti Sinha, Pooja Saraf

**Affiliations:** 1 Internal Medicine, Center for Diabetes and Internal Medicine, Delhi, IND; 2 Internal Medicine, Jaipur Golden Hospital, Delhi, IND; 3 Internal Medicine, Safdarjung Hospital, Delhi, IND

**Keywords:** hospital stay, sepsis, culture-positive, culture-negative, biomarker

## Abstract

Objectives: Sepsis is one of the leading causes of morbidity and mortality worldwide, and culture-negative sepsis, despite its prevalence, is largely understudied. The current study intends to examine clinical characteristics and biomarkers in culture-positive and culture-negative sepsis, focusing on 30-day mortality and duration of hospital stay in both groups.

Materials and methods: A prospective observational comparative cohort study was done on 150 patients admitted to the intensive care unit (ICU) and wards of Jaipur Golden Hospital. Patients with documented fungal, viral, or parasitic infections, as well as those who had undergone surgery or experienced trauma, were excluded.

Results: The mean age of the patients was 51.31±18.94 years. Of 150 patients, 95 (63.3%) were culture-negative, whereas 55 (36.7%) were culture-positive, with more men in the former and more women in the latter. Patients with negative cultures had fewer comorbidities. The levels of procalcitonin (PCT), C-reactive protein (CRP), and serum lactate were within the prescribed limit for both culture-negative and positive patients.

A higher proportion (87.3%) of the organisms isolated from culture-positive individuals were gram-negative, with *Escherichia coli* (*E. coli*) having the highest prevalence (27.3%), followed by *Klebsiella* (20%). There were 12.7% gram-positive isolates. The culture-negative patients had significantly better outcomes (P=0.003) as well as the duration of hospital stay (P<0.001) than the culture-positive patients. Culture-positive patients had a more severe illness, a higher incidence of septic shock, and a higher fatality rate than culture-negative patients.

Conclusion: It can be concluded that CRP and PCT can be used as clinically reliable sepsis biomarkers in both culture-positive and culture-negative patients. The study found that culture-negative sepsis is more prevalent and that there are substantial differences between culture-negative and culture-positive sepsis, with the former group having fewer comorbidities, less severe illness, a shorter duration of hospital stays, lower death rates, and better outcomes.

## Introduction

The global prevalence of septic shock and sepsis has grown over the past decade, and the related morbidity and fatality rates remain unacceptably high [1]. The death rate ranges from 21% to 85%, depending on the sources of infection [2]. Sepsis is defined as a dysregulated immunological response to an infection that can cause serious organ dysfunction [3]. The clinical criteria for diagnosing sepsis include a suspected or confirmed infection and a sudden increase in two or more sequential organ failure assessment (SOFA) scores that evaluate organ functioning [4]. Sepsis can be caused by a variety of microorganisms, although only a small number of these instances have been documented [5]. Combining the assessment of the systemic inflammatory response syndrome (SIRS) with the likelihood of infection creates a more complete strategy for recognizing and managing sepsis [6]. Bacteria are the primary cause of sepsis, and prompt management with suitable antibiotics can improve the chances of recovery [7]. A blood culture test, especially before antibiotic administration, is an essential aspect of treating individuals with sepsis [5]. There is little information on the characteristics and clinical outcomes of septic shock patients with positive or negative cultures. Sepsis or septic shock patients with negative cultures may exhibit unique changes in pathophysiology, epidemiology, and treatment responses as compared to those with positive cultures. There is a significant disparity in clinical findings between individuals with culture-negative and culture-positive [8], hence this area warrants additional investigation.

Biomarkers such as CP and PCT have been studied for a variety of uses in sepsis patients, including determining infections, predicting outcomes, and aiding with treatment decisions. These biomarkers provide valuable information for clinical decision-making and improving patient care. [9]. PCT is a thyroid hormone that increases with severe infections as well as cases of sepsis [10]. As a result, a rise in PCT levels in these patients may indicate a bacterial infection [11]. It is frequently employed to distinguish between bacterial infections, non-bacterial infections, and other inflammatory conditions [12]. CRP is a protein that reacts rapidly to acute events and shows interaction with the capsule C polysaccharides of *Streptococcus pneumoniae* [13]. Furthermore, measuring serum lactate levels is frequently used in the clinical care of severely ill patients, particularly in situations of septic shock and severe sepsis. It is frequently used to assess the severity of sepsis, gauge the efficacy of treatment, and predict outcomes [14,15]. An elevated white blood cell count (WBC) is also considered a key indicator of infection to diagnose sepsis. A higher WBC count indicates that the body is actively fighting against invading microorganisms, which aids in the diagnosis of infection [16]. It is crucial to understand that sepsis biomarkers are imperfect and may not always offer an accurate diagnosis. To achieve an accurate diagnosis, a thorough patient examination, as well as additional diagnostic procedures, should be done in combination with sepsis biomarkers. The purpose of this study was to examine and compare the clinical characteristics of individuals with culture-negative and culture-positive sepsis, as well as to see how effectively biomarkers predict sepsis outcomes. The study also sought to investigate the patients' 30-day mortality rate and duration of hospital stay.

## Materials and methods

Study design

**Figure 1:**
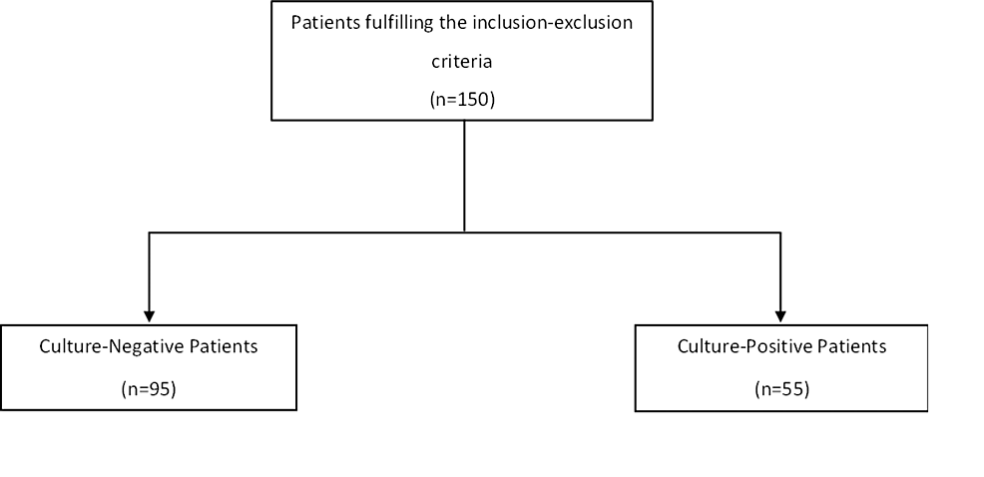
Distribution of the study population

This prospective, observational, comparative cohort study was undertaken at Jaipur Golden Hospital in New Delhi from September 2016 to May 2018. The study was granted permission by the research ethics and scientific committee of the institution. Patients ≥18 years of age with a body temperature > 38.0°C (hyperthermia) or < 36.0°C (hypothermia), heart rate (HR) > 90 beats per minute, respiration rate (RR) > 20 breaths per minute, and WBC count > 12,000 cells/mm^3^ or < 4,000 cells/mm^3^ were eligible for inclusion. Furthermore, patients were required to have substantial levels of biomarkers such as CRP > 0.5 mg/dl, PCT > 0.05 ng/ml, and serum lactate > 1.0 mmol/l. Finally, patients were included if they had a suspected or confirmed infection with at least one organ failure, were having hypotension and perfusion abnormalities despite adequate fluid resuscitation, or were using ionotropic or vasopressor medication. The exclusion criteria included patients with normal total leukocyte count (TLC), normal temperature, PCT levels < 0.05 ng/ml, CRP levels between 0 and 0.5 mg/dl, serum lactate levels between 0.4 and 1 mmol/l, trauma or burns, diabetes, fungal, parasitic, or viral infections, or previous surgery. Participants who met the inclusion and exclusion criteria and gave informed consent to participate in the study were enrolled (Figure 1).

Diagnostic criteria

In patients with documented or suspected infections, the diagnostic criteria included general variables such as fever (temperature higher than 38°C or lower than 36°C), elevated HR (above 90 beats per minute), altered mental status, tachypnoea (respiratory rate >20 breaths/min), pedal edema, or positive fluid balance >20ml/kg over 24 hours (fluid intake exceeds the output), and hyperglycemia (plasma glucose >140mg/dl) in the absence of diabetes. The existence of an infection and the body's systemic inflammatory response to the infection were determined by the levels of leucocytosis, leukopenia, WBC count, CRP, PCT, and serum lactate. The microorganisms were identified based on their respective cultures, which included blood, urine, or sputum, as per presentation. In addition to inflammatory markers, hemodynamic factors were employed to identify sepsis. Low systolic blood pressure (BP) or mean arterial pressure indicated hemodynamic instability, which might be attributed to the body's systemic response to an infection. The presence of organ dysfunction signs such as acute oliguria (urine output <0.5ml/kg/hr for at least 2 hours), rise in creatinine levels (>0.5mg/dl), coagulation abnormalities (INR >1.5 or a PTT>60 secs), ileus (refers to the intolerance of oral intake due to inhibition of the gastrointestinal propulsion without signs of mechanical obstruction)-absent bowel sound, thrombocytopenia (platelet count <150,000 cells/mm^3^), and hyperbilirubinemia (plasma total bilirubin >4mg/dl) played a major role in the diagnosis of sepsis. Hyperlactatemia, or elevated lactate levels (>1mmol/l) in the blood, was also a key tissue perfusion variable of sepsis.

Statistical analysis

Statistical testing was performed using SPSS Inc. Released 2008. SPSS Statistics for Windows, Version 17.0. Chicago: SPSS Inc. Continuous variables were reported as mean ± SD. Categorical variables were reported using frequencies and percentages. The student’s t-test was used to compare normally distributed continuous variables between the groups, whereas the Mann-Whitney U test was used to compare non-normally distributed continuous variables. The nominal categorical data was compared between the groups using the Chi-square or Fisher's exact test. A significant difference was considered in all statistical tests when a p-value of less than 0.05 was detected.

## Results

A total of 150 patients who met the inclusion-exclusion criteria were recruited for the study. Table 1 presents the clinical characteristics of the study population based on their culture results.

**Table 1:** Clinical characteristics of the study population * Significant value, statistical test: The Student’s t-test (for normally distributed continuous variables) or Mann-Whitney U test (for non-normally distributed continuous variables), whichever was applicable. The nominal categorical data was compared between the groups using the Chi-square or Fisher's exact test. SD: Standard deviation, SPO2: Oxygen saturation, BP: Blood pressure, RR: Respiratory rate

Characteristics	Culture Negative (N=95)	Culture Positive (N=55)	P-value
Age, Mean ± SD, years	51.31 ± 18.94	51.31 ± 18.94	0.649
Gender, n (%)			
Female	42 (44.2)	35 (63.6)	0.022*
Male	53 (55.8)	20 (36.4)	
Fever, n (%)	90 (94.7)	52 (94.5)	0.069
Cough, n (%)	21 (22.1)	11 (20.0)	0.725
Breathlessness, n (%)	21 (22.1)	12 (21.8)	0.421
Chest pain, n (%)	14 (14.7)	5 (9.1)	0.176
Dysuria/Burning Micturition, n (%)	16 (16.8)	20 (36.4)	0.012*
Vomiting/Nausea, n (%)	55 (57.9)	34 (61.8)	0.046*
Diarrhea, n (%)	18 (19)	6 (10.9)	0.033*
Dehydration, n (%)	31 (32.6)	7 (12.7)	0.025*
Pain abdomen, n (%)	48 (50.5)	23 (41.8)	0.223
Headache, n (%)	5 (5.3)	4 (7.3)	0.783
Neck rigidity, n (%)	4 (4.2)	4 (7.3)	0.465
Sensorium, n (%)	23 (24.2)	24 (43.6)	0.022*
Ulcers/bed sores, n (%)	11 (11.6)	11 (20.0)	0.160
Pedal edema, n (%)	19 (20.0)	18 (32.7)	0.081
Urine output (ml/kg), n (%)	15 (15.8)	16 (29.1)	0.071
BP, n (%)			
≥100/60-140/90 mmHg	58 (61.1)	29 (52.7)	0.035*
≤90/60 mmHg	27 (28.4)	11 (20.0)	
Not recordable	3 (3.2)	8 (14.5)	
≥150/80 mmHg	7 (7.4)	7 (12.7)	
Tachycardia (Pulse rate ≥ 90 beats/min), n (%)	88 (92.6)	53 (96.4)	0.050
Tachypnoea (RR >20 breaths/min), n (%)	23 (24.2)	7 (12.7)	0.090
Hypoxemia (SPO2 <90%), n (%)	12 (12.6)	3 (5.5)	0.258
Pre-existing illness (%)	42 (44.2)	32 (58.2)	0.099

In the current study, 95 (63.3%) patients had culture-negative sepsis, while 55 (36.7%) had culture-positive sepsis. Males outnumbered females in the culture-negative group, whereas the reverse was true in the culture-positive group (P=0.022). Out of 150 patients, 87 (58%) had BP in the range of ≥100/60 to 140/90 mmHg, 14 (9.3%) had BP ≥150/80 mmHg, 38 (25.3%) had BP ≤90/60 mmHg, and 11 (7.3%) had non-recordable BP when they were admitted. There was a strong association between high BP and culture positivity (P=0.035). Thirty individuals (20%) were found to have tachypnea with an RR of more than 20 breaths per minute. Out of these 30 patients, 23 (24.2%) had negative cultures, and seven (12.7%) had positive cultures. The study found no significant relationship between tachypnoea and the existence of positive cultures (P=0.090).

Table 2 presents the lab parameters of the study population.

**Table 2:** Lab parameters of the study population * Significant value, Statistical test: The Student’s t-test (for normally distributed continuous variables) or Mann-Whitney U test (for non-normally distributed continuous variables), whichever was applicable. The nominal categorical data was compared between the groups using the Chi-square or Fisher's exact test. CBC: Complete blood count, CRP: C-reactive protein, INR: International normalized ratio, KFT: Kidney function test, LFT: Liver function test, PCT: Procalcitonin, PT: Prothrombin time, RBS: Random blood sugar, TLC: Total leukocyte count Reference ranges: PCT: <0.05 ng/ml, CRP: <1.0 mg/dl, Serum lactate: <2 mmol/L, TLC: 4000 to 12000 cells/mm3, Platelets: 150000 to 400000 platelets/mcL, Total bilirubin (LFT): 0.1 to 1.2 mg/dl, Creatinine (KFT): 0.7 to 1.3 mg/dl, PT: 11 to 13.5 seconds, INR: 0.8 to 1.1, RBS: <140 mg/dl

Parameters	Culture Negative (N=95)	Culture Positive (N=55)	P-value
PCT >0.05 ng/ml, n (%)	95 (100.0)	55 (100.0)	-
CRP >0.5 mg/dl, n (%)	95 (100.0)	55 (100.0)	-
Serum lactate (%) >1 mmol/L	95 (100.0)	55 (100.0)	-
CBC, n (%)			
TLC <4000 or >12000 cells/mm^3^	86 (90.5)	42 (76.4)	0.018*
TLC raised with thrombocytopenia	9 (9.5)	13 (23.6)	
Deranged LFT, n (%)	11 (11.6)	23 (41.8)	0.873
Deranged KFT, n (%)	25 (26.3)	29 (52.7)	0.001*
Deranged PT/INR, n (%)	16 (16.8)	17 (30.9)	0.045*
RBS >140mg/dl, n (%)	23 (24.2)	19 (34.5)	0.174

All the patients that were included in the study had elevated PCT levels (higher than 0.05 ng/mL), suggesting the presence of bacterial infections. Of the 95 culture-negative patients, 86 (90.5%) were found to have TLC values less than 4,000 cells/mm^3^ or greater than 12,000 cells/mm^3^. However, none of these patients were immunocompromised. Nine patients (9.5%) had elevated TLC levels, thrombocytopenia, and a platelet count below 100,000 cells/mm^3^. Among the individuals with positive cultures, a greater proportion (23.6%) had both elevated TLC and thrombocytopenia than the culture-negative group (P=0.018). Forty-two patients (76.4%) in the culture-positive group had just elevated TLC levels. The percentage of culture-negative individuals with abnormal liver function tests (LFT) was 11.6%. In contrast, 23 patients (41.8%) who had elevated LFT tested positive for culture (P=0.873). These findings indicate a possible link between sepsis and liver impairment, with culture-positive sepsis having a greater impact on liver function. In the culture-negative group, 25 patients (26.3%) had raised creatinine levels, but in the culture-positive group, 29 (52.7%) patients had elevated creatinine levels (P=0.001). These findings indicate a greater frequency of altered kidney function tests (KFT) in individuals with culture-positive sepsis. In the culture-negative group, 16 (16.8%) patients had deranged PT/INR (Prothrombin/International normalized ratio). Whereas among culture-positive patients, 17 (30.9%) patients had a deranged PT/INR (P=0.045).

The culture positivity status (blood, urine, or sputum as per clinical presentation) of the study population is depicted in Table 3.

**Table 3:** Distribution of culture positivity among study population

Description	N = 55
Gram Negative Organisms, n (%)	48 (87.3)
E. coli	15 (27.3)
Klebsiella	11 (20.0)
Pseudomonas	7 (12.7)
Acinetobacter	4 (7.3)
Salmonella typhi	4 (7.3)
Enterococcus	4 (7.3)
Proteus	3 (5.4)
Gram Positive Organisms, n (%)	7 (12.7)
Streptococcus	5 (9.1)
Staphylococcus	2 (3.6)

Of the 55 culture-positive patients, 48 (87.3%) were gram-negative organisms, whereas seven (12.7%) were gram-positive organisms. Table 4 presents the duration of hospital stays and the outcome of the study population.

**Table 4:** Duration of hospital stay and outcome of the study population * Significant value, Statistical test: The Student’s t-test (for normally distributed continuous variables) or Mann-Whitney U test (for non-normally distributed continuous variables), whichever was applicable. The nominal categorical data was compared between the groups using the Chi-square or Fisher's exact test. LAMA: Left against medical advice

Parameters	Culture Negative (N=95)	Culture Positive (N=55)	P-value
Duration of hospital stay (%)			
0-7 Days	58 (61.1)	11 (20.0)	<0.001*
8-14 Days	34 (35.8)	38 (69.1)	
15-21 Days	3 (3.2)	5 (9.1)	
22-28 Days	0 (0.0)	1 (1.8)	
Cultures and outcome (%)			
Discharged	88 (92.6)	40 (72.7)	0.003*
LAMA	2 (2.1)	2 (3.6)	
Expired	5 (5.3)	13 (23.6)	

There was a strong association between culture positivity and mortality (23.6% vs. 5.3%, P=0.003), as well as duration of hospital stay (P<0.001).

## Discussion

The purpose of this study was to investigate and compare the clinical characteristics and biomarker levels of individuals diagnosed with sepsis based on whether their cultures were positive or negative. Biomarkers play a significant role in identifying and assessing sepsis because they demonstrate the absence, presence, or severity of bacterial, viral, and fungal infection, as well as the distinction between systemic sepsis and local infection [17,18]. In the clinical care of critical diseases, notably severe sepsis, and septic shock, we have explored serum lactate measurement in conjunction with PCT and CRP to reinforce our objective of comparison. In this study, all patients, whether culture-positive or culture-negative, had PCT >0.05 ng/mL, CRP >0.5 mg/dl, and serum lactate (%) >1 mmol/L. The study found a substantial association between CRP and PCT in the context of sepsis. According to the most recent sepsis recommendations, a new criterion for clinically detecting septic shock is the persistence of blood lactate levels of more than 2 mmol/L.

In our study, 63.3% and 36.7% of patients had negative and positive cultures, respectively. The study's findings are consistent with a meta-analysis of 22,655 patients, which indicated that about 40.1% (9086/22655) of sepsis or septic shock patients had a culture-positive infection [19]. The study found that the most important predictor of culture-negative status was the use of antibiotics over the previous 48 hours. Furthermore, it showed that the proportion of sepsis or septic shock patients caused by atypical pathogens, such as fungal and viral infections, might be increasing [20-22]. Furthermore, evidence shows that culture-negative sepsis provides distinct diagnostic challenges for medical practitioners and microbiologists, raising concerns about the accuracy of sepsis classifications [19].

The demographic characteristics of our study cohort, including mean age and gender distribution, were consistent with previous studies [20]. There was a notable difference in gender distribution between the positive and negative culture groups in this study. Males were more likely to exhibit negative culture results, whereas females were more likely to exhibit positive cultures. A previous study showed that culture-negative patients were more likely to be young men [20]. Furthermore, subsequent research found that female patients were more likely to have culture-positive sepsis [8]. Additional research found that female patients were more likely to be culture-positive, even after accounting for other variables [20].

All the patients included in the study had a clear source of suspected infection. The microorganisms were identified based on their respective cultures, which included blood, urine, or sputum, as per presentation. In this study, we also found some notable symptom differences between the positive and negative culture groups, as more patients with positive cultures had sensorium, vomiting, and dysuria/burning micturition. On the other hand, a higher number of those with negative cultures showed symptoms such as dehydration and diarrhea. The findings are consistent with a previous study, which found that people with positive cultures were more likely to experience urinary symptoms, with the urinary tract being the most prevalent region among positive culture cases [8]. Furthermore, because the gastrointestinal tract was the most common site in negative culture cases, gastrointestinal symptoms may be more common in these individuals [8]. In this study, 61.1% of patients with a negative culture had normal BP (≥100/60-140/90 mmHg), 28.4% had hypotension (≤90/60 mmHg), and 7% had hypertension. Patients with a positive culture had a greater percentage of tachycardia (pulse rate > 90 beats/min) than those with a negative culture, although the difference was not statistically significant. A previous study also found that culture-positive patients had a higher HR (108 vs. 83, P<0.001) than culture-negative patients [7]. Sepsis is characterized by a RR of more than 20 breaths per minute [23]. In this study, more patients with negative cultures had tachypnea (RR of more than 20 breaths per minute). The findings are consistent with a previous study, which found that pathogen-positive patients had somewhat less tachypnoea than pathogen-negative patients [8].

The WBC results were consistent with a previous study, with a higher number of patients in the culture-negative group showing TLC in the range of <4000 or >12000 cells/mm^3^. Elevated TLC levels with thrombocytopenia were higher in the culture-positive group, which is consistent with a previous study demonstrating that patients with a positive culture often had higher infection indicators in their WBC counts (13.7 vs. 10.5, P<0.001) [7]. Previous studies have shown that a WBC count of more than 12x10⁹/L and less than 4x10⁹/L implies sepsis [23]. Furthermore, in this study, a higher proportion of patients with a positive culture had a significantly deranged PT/INR than patients with a negative culture. Studies in the past have indicated a higher rate (35.53%) for PT in positive blood cultures than in negative blood cultures (19.7%), with a significant association (P=0.033) [24]. We also found that a higher proportion of patients with a positive culture had a deranged LFT than patients with a negative culture. This is consistent with a previous finding that showed higher elevated bilirubin (1.3 vs. 0.9 mg/dL, P<0.01) in the culture-positive sepsis group than in patients with culture-negative sepsis [8].

Furthermore, gram-negative organisms were found in a higher proportion of patients (87.3%) with positive cultures than gram-positive organisms (12.7%). These findings are consistent with a previous study, which indicated that gram-positive bacteria were less prevalent in patients with culture-positive sepsis than gram-negative bacteria [7,8]. Another study found that 25.67% and 38.96% of the study population had gram-positive and gram-negative bacterial infections, respectively [25]. Furthermore, in this study, we found that *E. coli* (27.3%) was the most common causative organism, followed by *Klebsiella* species (20.0%), *Pseudomonas* (12.7%), *Acinetobacter*, *Salmonella typhi* and *Enterococcus* (7.3%), and *Proteus* (5.4%), whereas *Streptococcus* (9.1%) was the most common in gram-positive organisms, followed by *Staphylococcus* (3.6%). These findings for gram-negative bacteria are consistent with past studies, which found that *E. coli* (37.3%) was the most prevalent causative organism, followed by Klebsiella species (19.8%) and others [7,8]. However, in the case of gram-positive bacteria, *Staphylococcus* (8.2%) was the most prevalent, followed by *Streptococcus* (6.2%), which contradicts our findings [8].

In this study, patients with a positive culture spent more time in the hospital than patients with a negative culture. Furthermore, a smaller number of patients with positive cultures were discharged from hospitals, and they also had a higher death rate compared to those with negative cultures. A meta-analysis found that the culture-positive group had a longer hospital stay than the culture-negative group (MD = -3.48; 95% CI, -4.34 to -2.63; P<0.00001; χ²= 1.03; I2 = 0%) [19]. Other studies also revealed that culture-positive patients had a longer post-sepsis length of stay (4.2 ± 5.9 days vs. 3.5 ± 5.1, P<0.001) and greater mortality (16% vs. 12%, P<0.001) [26]. The study found that these disparities are most likely caused by variations in patient demographics, infection location percentages, and antibiotic-resistant bacteria [19]. However, a meta-analysis found no significant difference in ICU length of stay between the two groups (MD = -0.19; 95% CI, -0.42 to 0.04; P=0.10; χ² = 5.73; I2 = 48%) [19]. 

This study has a few limitations that should be discussed. This is a single-center study conducted by an institution, which may restrict the extent to which the findings may be applied more generally. The study divided sepsis into two basic categories based on the presence or absence of pathogenic microorganisms; however, both groups are likely to be heterogeneous in terms of diagnosis. The study employed a three-reviewer adjudication approach to evaluate the results, although diagnostic misclassification remains a possibility. The findings may have been different if a larger sample had been analyzed and alternative causes of illness had been found. The markers' levels were based on hospital references, and because the study was observational, uncorrected confounders might have altered the data and conclusions.

## Conclusions

In conclusion, a large population of patients with sepsis was studied, and gram-negative microorganisms were the most commonly identified organisms. CRP and PCT were clinically reliable sepsis biomarkers in both culture-positive and culture-negative patients. Patients with negative cultures had a lower mortality rate and a shorter duration of hospital stay compared to those with positive cultures. Septic shock and death were more common in culture-positive individuals. Further research is needed to better understand the underlying causes of sepsis in culture-negative individuals.
